# BabyWASH and diarrhea prevention practices following multimedia educational intervention in hard-to-reach areas of the Afar and Somali regions of Ethiopia: a mixed-method endline evaluation

**DOI:** 10.1186/s12889-023-16887-y

**Published:** 2023-10-13

**Authors:** Abel Negussie, Ephrem Lejore, Ariam Hailemariam, Bereket Tefera, Elyas Melaku Mazengia, Tariku Dejene, Yared Tadesse, Yimenu Adane, Kalkidan Gugsa, Kabuka Banda, Rachana Sharma, Eshetu Girma

**Affiliations:** 1 Department of Social and Population Health, Yirgalem Hospital Medical College, Yirgalem, Ethiopia; 2 School of Public Health, College of Medicine and Health Sciences, Hawassa University, Hawassa, Ethiopia; 3 School of Medicine, College of Health Sciences, Addis Ababa University, Addis Ababa, Ethiopia; 4 Department of Public Health, College of Medicine and Health Sciences, Dire Dawa University, Dire Dawa, Ethiopia; 5 Department of Public Health, College of Health Sciences, Debre Markos University, Debre Markos, Ethiopia; 6 Center for Population Studies, College of Development Studies, Addis Ababa University, Addis Ababa, Ethiopia; 7 Ministry of Health, Addis Ababa, Ethiopia; 8 Social and Behavior Change (SBC) Section, United Nations Children’s Fund, Addis Ababa, Ethiopia; 9 Water, Sanitation and Hygiene (WASH) Section, United Nations Children’s Fund, Addis Ababa, Ethiopia; 10 School of Public Health, College of Health Sciences, Addis Ababa University, Addis Ababa, Ethiopia; 11 Ethiopian Health Education and Promotion Professionals Association (EHEPA), Addis Ababa, Ethiopia

**Keywords:** BabyWASH practices, Diarrhea prevention, Multimedia educational intervention, Effectiveness, Potential sustainability, Endline evaluation, Ethiopia

## Abstract

**Background:**

Water, sanitation, and hygiene (WASH) interventions, which are specifically targeted towards young children—known as “BabyWASH”—reduce exposure to environmental contamination and prevent microbial burden in their play and feeding environments. The purpose of this endline study was to evaluate the effectiveness and potential sustainability of a multimedia educational intervention in influencing key BabyWASH and diarrhea prevention practices in four hard-to-reach woredas (i.e. administrative districts) of the Afar and Somali regions of Ethiopia.

**Methods:**

A mixed-method, comparative cross-sectional study was conducted, which included 457 household surveys, 16 key informant interviews, and 8 focus group discussions. The multimedia educational intervention comprised: broadcasting radio talk shows and radio spot messages, capacity-building training for community health workers and community leaders, community mobilization campaigns, and the distribution of promotional print media materials. Propensity score matching analysis was used to estimate the effect of the multimedia educational intervention on key BabyWASH and diarrhea prevention attitudes and practices, which was then triangulated with qualitative findings.

**Results:**

The multimedia intervention had a significant positive impact on good BabyWASH and diarrhea prevention practices, including appropriate practices of child feces disposal (t-test = 5.17; p < 0.001), handwashing with soap or ash (t-test = 8.85; p < 0.001), maintaining separate playgrounds for young children (t-test = 2.83; p < 0.001), washing of child’s body, hands, and faces (t-test = 15.78; p < 0.001), and food hygiene practices (t-test = 2.74; p < 0.05). The findings of the qualitative assessment also revealed that the multimedia intervention packages and the approaches used were successful in influencing key BabyWASH and diarrhea prevention behaviors in the intervention implementation woredas. In addition, providing capacity building training to local actors and community leaders and recording radio talk shows and sharing them with community members were recognized as effective intervention implementation strategies.

**Conclusion:**

The endline evaluation found that the multimedia educational intervention improved awareness, perception, and practice of BabyWASH and diarrhea prevention behaviors in intervention woredas compared to control woredas. Sanitation and hygiene promotion interventions in pastoralist settings can be effective when using locally and contextually appropriate intervention strategies. However, considerations for integrating both behavioral and structural components in WASH interventions is essential.

**Supplementary Information:**

The online version contains supplementary material available at 10.1186/s12889-023-16887-y.

## Background

For many young children in low-resource contexts, the environment poses a substantial microbiological threat that may impact their health and development [1–3]. Due to the frequent interactions between children and their environments and the potential for repeated diarrheal illnesses in the first two years of life, water, sanitation, and hygiene (WASH) are crucial for children [2]. Through exploratory play and mouthing behaviors such as putting hands, objects, and soil into mouths in a contaminated domestic environment with animal and human feces, infants and young children can be exposed to microbial ingestion and subsequent health risks [1, 4]. Ethiopia is among the top five countries where diarrhea-related deaths are most common [5, 6], and the pooled prevalence of diarrhea among under-five children was also found to be 22% [7].

While there has been improvement in the WASH status in low- and middle-income countries (LMICs) [8], most mothers and caregivers lack knowledge about proper management of child feces and environmental health risks that infants and young children are exposed to [1, 9]. Similar to other LMIC settings, there is a common misconception in Ethiopia that “children’s feces are not harmful to health” [10]. According to a recent study report, 25% of children’s feces in Ethiopia are either left in the open or are not properly disposed of [11]. Improper child feces management, however, may be a significant source of exposure, as young children have the highest incidence of enteric infections and their feces are most likely to contain higher pathogenic loads [12, 13]. Moreover, poor child feces management practices, along with other key WASH-related factors such as limited access to water, poor sanitation and hygiene, and a lack of water treatment facilities, can increase the risk of childhood diarrheal diseases [13, 14].

As a result of the mixed findings on the effect of simple and basic WASH interventions on child health and development outcomes and with the recognition of the lack of a holistic view in WASH interventions [2, 15, 16], “BabyWASH” emerged as a new concept of WASH to target the WASH needs of children under the age of three years old [1, 6]. The aim of BabyWASH is to protect the health of infants and young children by decreasing exposure to microbial pathogens in their play and feeding environments. In 2017, the Ministry of Health of Ethiopia, supported by the United Nations Children’s Fund (UNICEF), developed a Baby and Mother WASH implementation guideline to raise public awareness [6]. The guideline describes a set of WASH interventions that focus on pregnant women, babies, and children under the age of three years. Areas covered in the guideline include child feces disposal, handwashing with soap, protective play, food hygiene, encouraging the containment or penning of animals in the home, and promoting the importance of shoes for children when they begin walking.

BabyWASH interventions may be effectively promoted using culture- and context-specific behavior change strategies. There are no “silver bullets” that apply to all settings equally, and each setting requires a unique intervention approach when implementing BabyWASH packages [2, 6, 17]. Noticeably, in pastoralist communities in Ethiopia, such as Afar and Somali, infants and young children regularly contact with livestock and their feces, and this poses a constant health risk. In pastoralist settings, the prevalence of diarrhea among children less than two years of age was found to be as high as 26–31%, which is larger than the national prevalence [7, 18, 19]. In addition, a BabyWASH baseline survey revealed that in rural pastoralist areas, only 38% of women have knowledge on the health risks of unsafe disposal of child feces, and only 22% of caregivers of young children under the age of three reported they wash their children’s bodies on daily basis [6]. There is also a paucity of evidence to support which WASH interventions are most relevant for diarrhea prevention in these pastoralist settings.

Evidence-based and context-specific behavioral interventions that can impact community-level knowledge, perceptions, and practices are required to effectively implement the BabyWASH components and inform best approaches [6]. In this regard, multimedia educational campaigns may impact BabyWASH-related outcomes [13, 20]. And research is needed to inform what social and behavior change strategies are effective at influencing BabyWASH behaviors, especially in underserved groups with higher risk of diarrhea incidence. The importance of embedding research into practice is also emphasized in the Ethiopia BabyWASH implementation guideline as there is still much to understand about behavior change strategies that are contextually appropriate and can be widely adapted to other specific target groups [6]. Therefore, the purpose of this endline study was to evaluate how much a multimedia educational intervention influenced mothers’/caregivers’ and other household and community members’ key BabyWASH and diarrhea prevention practices in hard-to-reach areas of the Afar and Somali regions of Ethiopia, as well as the extent to which the intervention outcomes likely sustain after the intervention.

## Methods

### Design and study site

For the endline evaluation study, a comparative cross-sectional design with a mixed-method of quantitative and qualitative approaches was employed. Geographically matched, hard-to-reach woredas (i.e. administrative districts) in the Afar and Somali regions were assigned as intervention and control woredas.

Four hard-to-reach woredas or districts of the Afar and Somali regions, Afdera and Elidar in the Afar region and Shinile and Gashamo in the Somali region, were selected as “Intervention Woredas,“ while another two woredas from each region, Kuri and Bidu woredas in the Afar region and Harshin and Errer woredas in the Somali region, were selected as “Control Woredas” (non-intervention woredas). The selection of intervention woredas was based on the presence of WASH programs, and the woredas are also targeted for WASH programs that include social and behavioral change interventions as part of the existing WASH program. On the other hand, the selection of control woredas was based on their similarity in terms of socioeconomic status, including educational status, employment status, and low access to radio and television, and livelihood system (i.e., pastoralist way of living), as indicated in the 2007 national census study, which was the most recent census report available at the time of data collection. As a result, the following commonalities between project intervention and control woredas have been considered: a large segment of the population is semi-pastoralist, illiterate, economically active and has similar trend of employment rate, and having similar clan-based system. Besides, better accessibility and geographical reachability have also been considered as criteria when selecting control woredas.

According to our formative assessment, both the intervention and control woredas in the Somali and Afar regions lack basic WASH access. There is a persistent water shortage in these woredas, and the majority of the community rely on unsafe tanker tracks and rainwater collection to meet their needs.

### Intervention description

Population Media Center Ethiopia in collaboration with UNICEF has implemented a multimedia educational intervention for one and a half years (July 2018 to March 2020) in four hard-to-reach woredas in the Afar and Somali regions. The multimedia educational intervention intended to promote mothers and caregivers to adopt appropriate BabyWASH and hygiene behaviors for children under three years of age as well as to improve desired changes for diarrhea prevention and control by influencing potential behavioral determinants such as awareness and perceived benefits of good BabyWASH and diarrhea preventive practices, beliefs about the detrimental effects of poor BabyWASH target behaviors on child health, and perceptions of social norms.

The development of the educational intervention was informed by a qualitative formative study, which revealed that, while rural communities in Afar and Somali regions have high trust in electronic media such as radio and TV as reliable sources of information, they have low access. Many people in the study areas were also illiterate to access and obtain information from print media, and prefer pictorial posters or flyers that consider the cultural context of the community over written messages. Moreover, the formative assessment found that interactive approaches leveraging traditional music, poems, and dramas can be effective behavior change strategies for improving BabyWASH attitude and practice for a larger segment of the illiterate target communities. The key findings from the qualitative formative assessment also include that the impact of radio can be enhanced when people with similar interests listen to the radio in groups, as a radio program helps bring community together and encourages members and families of listeners’ groups to know more about recommended hygienic behaviors and practice and try new ways of life style that are related to BabyWASH. Thus, based on the formative research findings, the multimedia educational intervention was desired for its potential to reach a wide audience, cater to different learning styles for increasing the likelihood of BabyWASH behaviors adoption, and empower local communities.

In general, the proposed intervention assumed that the BabyWASH and diarrhea preventive behavioral targets could be achieved through locally appropriate and context-sensitive multimedia educational strategies, including: broadcasting radio talk shows and radio spot messages, providing Health Extension Workers (HEWs), who are frontline community health workers, and community leaders (local representatives, clan leaders) with capacity-building training, conducting community mobilization campaigns, and distributing promotional print media materials. Furthermore, “radio listener groups” that listen to the radio show off-air and facilitate group discussions following each radio program have been established. Importantly, intervention implementation strategies used include the use of government structures and WASH approaches consistent with regional and national WASH programs. Relevant local government sector offices, including health bureaus and offices at all levels also participated in the planning and implementation of key intervention components. A summary description of the different components of the multimedia educational intervention is presented as follows.

#### Radio talk shows

Radio talk shows have been broadcast in the form of radio magazines to raise public awareness, change attitudes, and promote BabyWASH and diarrhea prevention practices. The radio talk shows were produced in Afar and Somali languages: Hangi Ala’ah’ in Afar language means “focus on children,” and “Daryel” in Somali language means “care for children”. Additionally, the radio talk shows are built around interviews, book reviews, short plays, Vox pop (imparting ideas of different people on a single topic/issue), poems, narration, and features. Other multimedia communication approaches, such as radio serial drama, used to complement radio talk shows. As well, the messages conveyed during the radio talk shows emphasized addressing pertinent issues related to BabyWASH (i.e., safe child disposal, handwashing at critical times with soap/ash and creating safe environment/protective play to children under three years at household level) and diarrhea prevention including food hygiene, water treatment at household level, and personal hygiene with an emphasis on washing children’s body, face, and hands (Additional file 1). Sixty episodes of radio talk shows was broadcast for 30-minutes in every week through the Ethiopian Broadcasting Corporation (EBC), which is a national TV and radio broadcasting station.

A total of 39 “radio listener groups” (20 in the Afar region and 19 in the Somali region) have been set up, in which one group comprises 8–12 members. “Radio listener groups” are groups that were provided with a solar radio and a memory card to record and document all the sessions of the radio talk shows. They were also able to listen to the radio show off-air as most people in the study woredas don’t have access to radios. At the end of listening to each radio program, the radio listener groups expected to discuss, reach consensus, and reinforce or change other community members practices based on the learnings from the shows on the importance of BabyWASH and diarrhea prevention practices, especially for mothers/caregivers of children under three years of age. Membership was voluntary, and most radio listener groups have been homogenous, having independent male and female groups.

#### Broadcast of radio spot messages

Radio spot messages were produced and broadcast through radio for a maximum of 90 min. A series of key spot messages emphasizing BabyWASH and diarrhea prevention were frequently broadcast to a wider audience. Similar to the radio talk show programs, the spot messages have been transmitted in both the local languages of Afar and Somali. The program has made attempts to make the radio spot messages attention-grabbing using simple and plain languages, so that key messages on BabyWASH and diarrhea prevention remain memorable to larger audiences.

#### Community mobilization campaigns

Community mobilization campaigns were the other central strategy used to disseminate key messages to improve awareness and practice in the target community. The campaigns were conducted at social gatherings, festivities, occasions, and other events such as community health discussion forums. An estimated 82,937 people (Male – 44,205; Female – 38,732) in all intervention woredas were reached through the community mobilization campaigns. During these gatherings, practical handwashing demonstrations with soaps were conducted, and key messages on BabyWASH and diarrhea prevention were conveyed to the attendees by trained HEWs. In addition, a total of 4,840 potties and soaps (2,062 in Somali and 2,778 in Afar) were distributed to those attending the sessions.

#### Capacity-building training

In the intervention woredas in the Afar and Somali regions, capacity-building training was provided for 88 community representatives (Somali – 41, 34 male and 7 female; Afar – 47, 37 male and 10 female) and 29 journalists on general hygiene, BabyWASH, and diarrhea prevention. Similar to other intervention strategies, capacity-building training centered on raising community members’ awareness of the importance of BabyWASH and its association to health outcomes and early childhood growth and development. Community representatives were equipped with communication skills to effectively promote BabyWASH and diarrhea prevention target behaviors to parents, families and the community at large. The logic behind the efforts to enhance these community members’ capacities is that doing so would have a multiplier effect in educating and communicating crucial messages to the wider targeted communities on proper hygiene and sanitation practices in general and assert changes in BabyWASH practices in particular.

#### Print media production and dissemination

The multimedia intervention packages included print media (leaflets and posters) to disseminate messages regarding BabyWASH and diarrhea prevention to community members of the intervention woredas in various settings and platforms, including health bureaus and offices, schools, and during community mobilization events. The promotional print materials were prepared in local languages and also used to facilitate discussion in radio listener groups.

### Study participants

A household member, primarily a woman older than 18 years and preferably a mother of young children, participated as an informant in the household survey. The sample size was determined based on the following assumptions: a prevalence (p) of 50%, 95% confidence level, 5% margin of error, design effect of 1.2%, and 5% non-response rate. As the project lacked a baseline survey, the proportion of the target group washing their hands with soap/ash at critical times has been taken as 50% (p). Based on the above assumptions, the final sample size was 483.

A three-stage sampling method was used to select households from each study woreda. First, the calculated sample size was allocated for the study woredas using the probability proportional to size (PPS) technique based on the total population of the woredas. Then, two *kebeles* (lower administrative units) were selected from each woreda and the sample for each study woreda had been distributed to *kebeles*. Finally, households in each *kebele* that fulfilled the inclusion criteria—those with children under three years old—were randomly selected from HEW registrations.

### Data collection procedure

Quantitative and qualitative data collection took place concurrently between June 14 and June 27, 2020 in both the intervention and control woredas. A structured household survey questionnaire that was designed based on the BabyWASH program components and the intended target outcomes was administered using the Open Data Kit (ODK) platform. Due to the advantages of automating interviews, such as skip instructions, the usage of ODK helped avoid data entry errors and enabled to supervise data collection in a much more effective way.

The designed survey questionnaire was refined and improved with input from UNICEF WASH team staff. Based on cut-off measurement values suggested by expert desk review techniques, each key practice related to BabyWASH and diarrhea prevention was operationally categorized as “Good” and “Poor”. Mothers and women caregivers were given preference during data collection to act as primary respondents whenever they were available. The data were collected by highly experienced data collectors and research assistants who have received training and can speak local languages. Moreover, the data collection process was supervised by field supervisors to ensure data quality and that it was carried out in compliance with the approved planned methodology.

In order to substantiate the findings of the quantitative evaluation, qualitative data was collected through focus group discussions (FGDs), key-informant interviews (KIIs), human interest stories, and observation. Eight FGDs with a total of 16 homogeneous participants (20 females and 12 males) were held with radio listener groups and HEWs in the project intervention woredas. Because of COVID-19 restrictions at the time the data was collected, only 3–4 participants were included in each FGD. Additionally, 16 KIIs were conducted with stakeholders from the Woreda Health Office, HEWs, community mobilizers, and clan leaders. Topic guides were prepared based on the multimedia intervention components and key BabyWASH and diarrhea prevention target outcomes. A team of trained qualitative research assistants facilitated the qualitative data collection. Human interest stories—that is, stories of interesting personal experiences of the BabyWASH project’s direct beneficiaries—have also been gathered to gain a better insight of the intervention’s impact.

### Intervention outcomes and measures

The target BabyWASH and diarrhea prevention intervention outcomes include safe disposal of child feces (i.e., disposal of the child’s feces into the latrine), handwashing at critical times with soap/ash, protective play for children, shoe wearing behavior to a child under the age of three, food hygiene, safe water treatment at household level, and child hygiene practices (i.e., washing the body, hands and face of a child under the age of three). Salient BabyWASH and diarrhea preventive behaviors at the individual and community levels were compared between project intervention and control woredas using both qualitative and quantitative approaches. For the quantitative assessment of the effect of the BabyWASH and diarrhea prevention intervention, operationally categorized measurement cut-off points were applied to create dichotomous variables of “good” and “poor” practices (Additional file 2). Despite the relatively short duration of the intervention (1.8 months), this endline evaluation study also qualitatively explored the potential sustainability and issues seen as threats to the long-term sustainability of the intervention outcomes in project intervention woredas.

### Data analysis

The effectiveness of the multimedia educational intervention on key BabyWASH-related outcome changes was evaluated by triangulating quantitative and qualitative data from both the intervention and control woredas.

The survey data was exported from the ODK tool, and relevant descriptive statistics were computed using SPSS version 20. Results from the intervention and control woredas are compared using BabyWASH and diarrhea prevention target indicators. During data analysis, it was identified that 21 participants from the control woredas in the Somali region had exposure to the multimedia intervention through the radio talk show programs (locally known as “Daryel” in Somali), and they were thus excluded from the analysis. To specifically estimate the effect of the multimedia educational intervention on key BabyWASH and diarrhea prevention attitudes and practices, propensity score matching (PSM) analysis was employed by matching each participant from the intervention woreda with a participant of similar characteristics from the control woredas. Age, sex, and marital status were used as matching variables, and a t-test for independent proportion difference (difference-in-proportion) was employed to estimate the effects of the intervention. P-values less than 0.05 were considered as significant.

In addition, the qualitative data from multiple sources was transcribed, translated into English, summarized, and then analyzed using thematic analysis and multi-method triangulation techniques. An inductive, open thematic coding approach guided by the BabyWASH focus areas was followed to identify concepts and key themes. The analysis involved five sequential and linked iterative steps: open-minded reading and re-reading, coding, displaying, reducing, and finally interpretation of meanings in relation to the study topic [21]. The analysis focused on themes relating to level of the impact of the multimedia intervention, the ways in which the intervention packages influenced BabyWASH and diarrhea prevention behaviors, and the potential sustainability of intervention outcomes. Illustrative verbatim quotes have also been used where appropriate to support result descriptions.

## Results

### Sociodemographic characteristics of the household survey participants

A total of 457 households participated in the survey (95% response rate), and 21 participants from the control woredas were excluded from analysis since they had exposure to the particular multimedia intervention. Out of the total 436 eligible participants considered for the comparative analysis, 259 participants included from the intervention woredas in the Afar and Somali regions, while the other 177 participants included from the control woredas in the two regions. In this regard, 145 participants (33.3%) from the intervention and control woredas in the Afar region and 291 participants (66.7%) from the intervention and control woredas in the Somali region were involved.

The majority of survey participants (93.8%) were female, and 64% of them were mothers or caregivers of children under three who had one (66.1%) or two (33.9%) under-five children. Additionally, in both regions, the majority of survey participants were married (88.1%) and Muslims (98.6%). In terms of educational background, 46% of participants were illiterate (unable to read and write), 37% can read and write but hadn’t attended formal education, and only about 16% of the survey participants had attended formal education. The majority of households (73.9%) had 6–7 family members in both the intervention and control woredas of the two regions (Table 1).

**Table 1:** Sociodemographic characteristics of the household survey participants, N = 436

Variables	Survey participants		Total (N = 436) n (%)
	Intervention Woredas (N = 259) n (%)	Control Woredas (N = 177) n (%)	
Region			
Afar	87 (33.6)	58 (32.8)	145 (33.3)
Somali	172 (66.4)	119 (67.2)	291 (66.7)
Sex			
Female	246 (94.9)	163 (92.0)	409 (93.8)
Male	13 (5.0)	14 (7.9)	27 (6.2)
Age			
18–24	44 (16.9)	21 (11.9)	65 (14.9)
25–34	94 (36.3)	68 (38.4)	162 (37.2)
35–44	52 (20.0)	60 (33.9)	112 (25.7)
45 and above	69 (26.6)	28 (15.8)	97 (22.2)
Educational status			
Illiterate	121 (46.7)	82 (46.3)	203 (46.5)
Able to read & write	81 (31.3)	80 (45.2)	161 (36.9)
Primary education	45 (17.4)	15 (8.5)	60 (13.8)
Secondary education	9 (3.5)	0 (0)	9 (2.1)
College diploma	3 (1.1)	0 (0)	3 (0.7)
Marital status			
Married	222 (85.7)	162 (91.5)	384 (88.1)
Divorced	6 (2.3)	2 (1.1)	8 (1.8)
Widowed	13 (5.0)	9 (5.0)	22 (5.0)
Single	18 (6.9)	4 (2.3)	22 (5.0)
Household size			
4–5	50 (19.3)	36 (20.3)	86 (19.7)
6–7	192 (74.1)	130 (73.4)	322 (73.9)
8–10	17 (6.6)	11 (6.2)	28 (6.4)
Religion			
Muslim	253 (97.7)	177 (100)	430 (98.6)
Orthodox	6 (2.3)	0 (0)	6 (1.4)
Number of < 5 years children			
1	167 (64.5)	121 (68.4)	288 (66.1)
2	92 (35.5)	56 (31.6)	148 (33.9)

### Effectiveness of the intervention

The effectiveness of the multimedia educational intervention was evaluated in relation to influencing awareness, attitudes, and practices pertaining to key BabyWASH and diarrhea preventive behaviors in the targeted communities of the intervention vs. control woredas through the triangulation of quantitative and qualitative data on intervention outcomes. As a result, the intervention’s outcomes on knowledge, attitudes, and behavior regarding key BabyWASH and diarrhea prevention indicators have been examined and described.

### Effectiveness in raising awareness on key BabyWASH and diarrhea prevention behaviors

The effectiveness of the educational intervention, especially radio talk shows and community mobilization events, in conveying BabyWASH and diarrhea prevention messages to the community was assessed through analysis of the household survey data. The results showed that only 40 (22.6%) of respondents from the control woredas had information on safe disposal of child feces, compared to 226 (87.3%) of respondents from the intervention woredas, indicating that household respondents in the intervention woredas were more aware than respondents in the control woredas. Similarly, awareness of the importance of handwashing with soap/ash at critical times appeared to differ between participants from intervention and control woredas (Table 2).

**Table 2:** Household respondents’ awareness of key BabyWASH and diarrhea prevention behaviors in intervention vs. control woredas of Afar and Somali regions of Ethiopia, 2020

Awareness of key BabyWASH and diarrhea prevention behaviors	Survey participants	
	Intervention Woredas n (%)	Control Woredas n (%)
Safe disposal of child feces		
Yes	226 (87.3)	40 (22.6)
No	33 (12.7)	137 (77.4)
Importance of handwashing with soap/ash		
Yes	242 (93.4)	104 (58.8)
No	17 (6.6)	73 (41.2)
Containment/penning of animals in the home		
Yes	194 (74.9)	83 (46.9)
No	65 (25.1)	94 (53.1)
The importance of wearing shoes for children < 3 years age		
Yes	139 (53.7)	80 (45.2)
No	120 (46.3)	97 (54.8)
Preparing safe food in the household		
Yes	188 (72.6)	73 (41.2)
No	71 (27.4)	104 (58.8)
Safe water treatment at home before consumption		
Yes	232 (89.6)	79 (44.6)
No	27 (10.4)	98 (55.4)
Importance of washing under-three children’s bodies, hands, and faces		
Yes	213 (82.2)	78 (44.1)
No	46 (17.8)	99 (55.9)

**Fig. 1:**
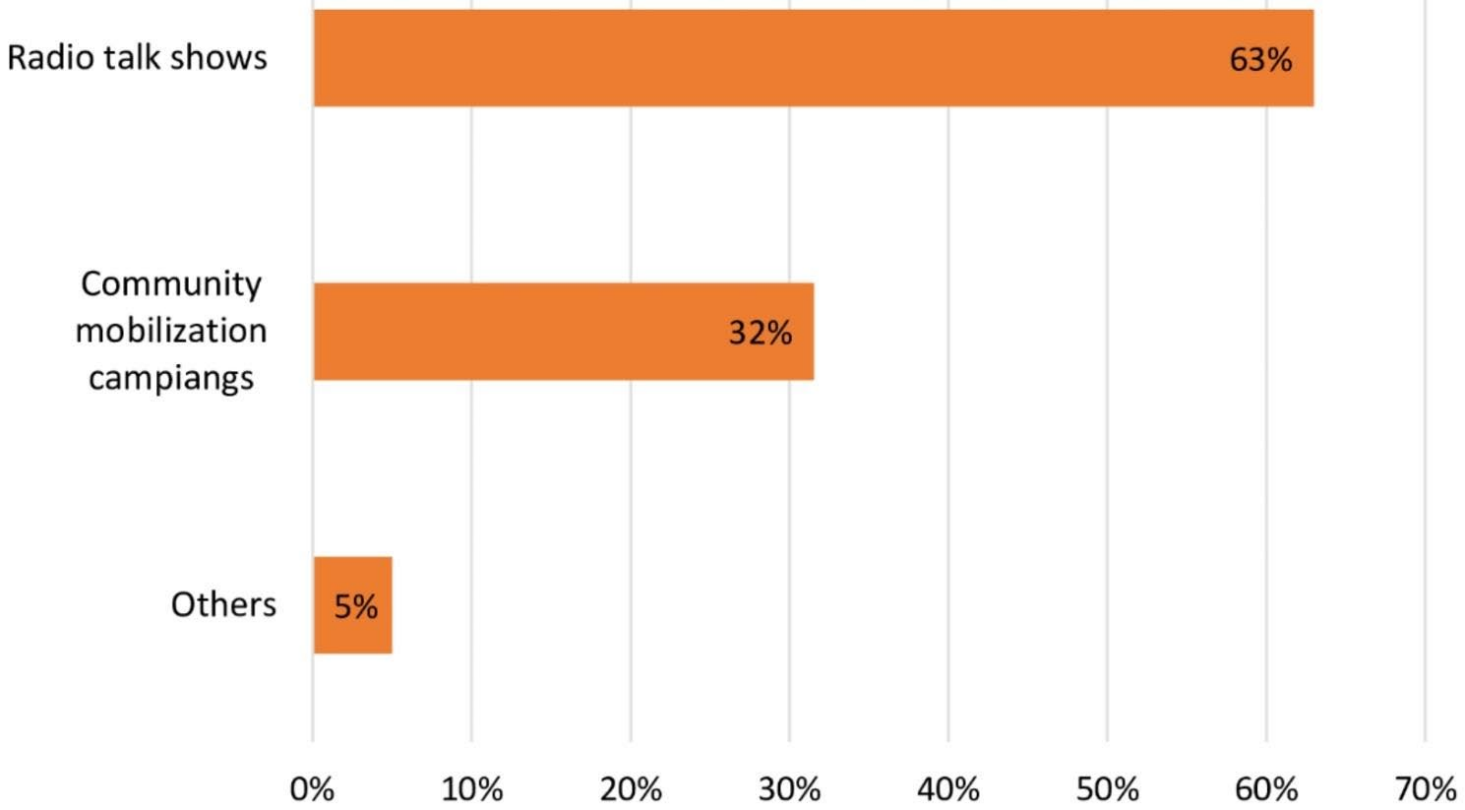
Proportion of survey participants having access to information on BabyWASH and diarrhea prevention by source of information in intervention woredas (N = 259)

Furthermore, it was found that the number of sample households in the intervention woredas that had information about containment/penning of animals in the home and maintaining an environment free from animal feces was 194 (75%), compared to less than half of the survey respondents in the control woredas. The respondents’ awareness of the importance of shoe wearing for children under three years of age, however, differs alike between intervention and control woredas. In addition, 232 (89.6%) and 213 (82.2%) respondents from the intervention woredas reported they had heard about safe water treatment at home before consumption and the importance of washing a child’s body, hands, and face, respectively, whereas only 44% of participants from the control woredas had information about these crucial BabyWASH and diarrhea prevention behaviors (Table 2). For the survey respondents from the intervention woredas, the sources of information on BabyWASH and diarrhea prevention were assessed. Radio was the most common source of information (63%), followed by community mobilization events (32%) (Fig. 1).

The results of the qualitative analysis also indicated that the radio talk shows and radio spot programs were the most common and intriguing sources of information for the BabyWASH and diarrhea prevention messages that were mostly frequently referred to by participants during interviews and group discussions. The radio listener groups especially emphasized that they had learned a lot from the radio program in which they regularly participated.

*“-----we learned through the radio shows how to protect children’s health and prevent diarrhea. First and foremost, the environment must be free from excreta, including child feces. People should not defecate in the open, but if they don’t have access to latrines, they should bury their feces instead” [FGD Participant, Radio Listener Group Member, Shinile Woreda, Somali Region]*.

Another FGD participant expressed that her involvement in the radio listener group helped her gain knowledge and make an effort to educate communities to bring about positive changes.

“*----Being a part of the radio listener group in our woreda has given me a lot of benefits, including learning practical household water treatment techniques such as boiling, filtering, or using chlorine------ I have also educated other community members who lack access to listen to the radio spots and talk shows-------” [FGD Participant, Radio Listener Group member, Afdera Woreda, Afar Region*].

The community mobilization campaigns may have been effective in conveying messages to a wide segment of the target audiences in intervention woredas. KII participants also appreciated the community campaign events carried out in the local languages.

*“------ I remember a campaign that took place a few months ago. It was a colorful event where people sang and danced to cultural music. At that event, a lot of information was disseminated, including the importance of handwashing with soap or ash and the setting up of a separate playground for babies at home. It was educational for mothers who had no previous knowledge of such health issues [BabyWASH and diarrhea prevention] --------” [KII Participant, Health Extension Worker, Elidar Woreda, Afar Region]*.

Furthermore, the importance of print media in educating mothers and other caregivers visiting medical facilities for various medical services was discussed during the interview with some HEWs. They asserted that the posters and leaflets disseminated to health facilities served as a basis for educating mothers/caregivers on issues related to BabyWASH and diarrhea prevention. In their view, HEWs stated that as the posters and leaflets were prepared using pictorial designs and local languages, they were easily comprehended by clients visiting health facilities and also helped illiterate mothers/caretakers in grasping the main messages of the posters and leaflets.

### Effectiveness in influencing beliefs and attitudes related to BabyWASH and diarrhea preventive behaviors

In contrast to respondents from the control woredas, where only 32 (18.1%) of the participants stated a positive belief about the proper disposal of child feces, 204 (78.7%) of the household respondents from the intervention woredas agreed that improper disposal of child feces can be harmful to health. Regarding their attitudes on the importance of maintaining an environment free of animal feces for the health of young children, there is also a marked percentage difference between intervention and control woredas respondents. It was found that 178 (68.7%) survey participants from intervention woredas agreed that maintaining an environment free of animal waste and dung was crucial for protecting children’s health, however, only 71 (40.1%) participants from the control woredas had this similar belief. As compared to respondents from control woredas, respondents from the intervention woredas had a more favorable attitude regarding treating household water before consumption and toward washing children’s hands, bodies, and faces, with percentages of 79% and 82%, respectively. However, only around half of participants from the intervention woredas agreed that it is vital for young children to wear shoes and that food hygiene is important for health, compared to 35% and 23% of participants from the control woredas, respectively (Table 3).

**Table 3:** Attitudes/beliefs related to key BabyWASH and diarrhea prevention target behaviors among survey participants in intervention vs. control woredas of Afar and Somali regions of Ethiopia, 2020

Attitude/beliefs related to key BabyWASH and diarrhea prevention behaviors	Survey participants		Propensity score matching analysis	
	Intervention Woredas n (%)	Control Woredas n (%)	Standardized proportion difference (95% CI)	T-test
A child feces is hazardous to health				
Agree	204 (78.7)	32 (18.1)	0.57 (0.35, 0.78)	3.40**
Disagree	55 (21.2)	145 (81.9)		
Maintaining an environment free from animal feces can protect the health of young children				
Agree	178 (68.7)	71 (40.1)	0.12 (-0.31, 0.21)	0.77
Disagree	81 (31.2)	106 (59.8)		
Hand washing with soap/ash is important to health				
Agree	209 (80.7)	87 (49.1)	0.12 (-0.17, 0.42)	0.75
Disagree	50 (19.3)	90 (50.8)		
Young children should wear shoes to protect them from any potential harm				
Agree	132 (50.9)	63 (35.6)	0.05 (-0.31, 0.21)	-0.30
Disagree	127 (49.1)	114 (64.4)		
Food hygiene is important to health				
Agree	139 (53.6)	41 (23.1)	0.03 (-0.18, 0.26)	0.24
Disagree	120 (46.3)	136 (76.8)		
It is necessary to treat drinking water at home before consumption				
Agree	205 (79.1)	63 (35.6)	0.21 (-0.04, 0.48)	1.29
Disagree	54 (20.8)	114 (64.4)		
Young children can be protected from disease by regularly washing their hands, bodies, and faces				
Agree	213 (82.2)	54 (30.5)	0.34 (0.10, 0.58)	2.09*
Disagree	46 (17.7)	123 (69.5)		

**P < 0.001 *P < 0.05

The PSM analysis demonstrated that the difference-in-proportion between the intervention and control groups on attitudes and beliefs related to child feces disposal and washing child’s body, face, and hands outcomes was statistically significant. The proportion of participants from the intervention woredas who have a favorable attitude towards the potential health risks of improper child feces disposal was more than three times higher than the proportion of participants from the control woredas (t-test = 3.4; p < 0.001). Likewise, the proportion of participants from the intervention woredas who have a favorable attitude towards the importance of washing the child’s body, hands, and face was two times higher than the proportion of participants from the control woredas (t-test = 2.09; p < 0.05) (Table 3).

Besides the survey results, the qualitative study findings provided evidence in support of the contribution multimedia intervention components made to addressing factors that affect BabyWASH and diarrhea prevention practices. The radio talk shows and radio spot messages broadcast, according to members of the radio listener groups, were a powerful tool for addressing key beliefs and attitudes pertaining to BabyWASH and diarrhea prevention. Based on the major issues raised during the discussions with radio listener group members who attended the radio-listening sessions, the contributions of radio talk shows and spots were especially influential in changing widespread beliefs and attitudes related to BabyWASH and diarrhea prevention behaviors.

*“-----the radio talk show programs discuss different topics, including safe child feces disposal. Before we heard on the radio how child feces could be hazardous to health if not safely disposed of into a latrine, mothers like myself did not think of child feces as harmful to health------” [FGD Participant, Radio Listener Group member, Afdera Woreda, Afar Region]*.

### Effectiveness in improving key BabyWASH and diarrhea prevention practices

The practice-level comparative differences between participants from intervention and control woredas with regard to BabyWASH and diarrhea prevention indicator variables were also examined using the household survey data. As a result, households’ practices in intervention woredas improved in terms of most BabyWASH and diarrhea prevention indicators when compared to control woredas, which were generally at a low practice level. For instance, the proportion of households that reported proper disposal of child feces (into latrine or buried) was nearly 70% in the intervention woredas while only 3% of the households in the control woredas were practicing proper disposal of child feces (Table 4). Likewise, there appeared to be a difference between respondents in the intervention and control woredas regarding practices of handwashing with soap/ash at most critical times as well as washing of a child’s body, hands and face. However, only 22 (8.5%) of respondents from the intervention woredas reported treating drinking water at the household level (Table 4).

**Table 4:** Key BabyWASH and diarrhea prevention practices among households in intervention vs. control woredas of Afar and Somali regions of Ethiopia, 2020, N = 436

Key BabyWASH and diarrhea prevention practices	Survey participants		Propensity score matching analysis	
	Intervention Woredas n (%)	Control Woredas n (%)	Standardized proportion difference (95% CI)	T-test
Proper child feces disposal				
Good	181 (69.9)	6 (3.4)	0.41 (0.31, 0.51)	5.17**
Poor	78 (30.1)	171 (96.6)		
Handwashing practices at critical times with soap/ash				
Good	190 (73.4)	4 (2.3)	0.70 (0.61, 0.78)	8.85**
Poor	69 (26.6)	173 (97.7)		
Maintaining separate playground for under-three years children				
Good	199 (76.8)	46 (26.0)	0.39 (0.22, 0.55)	2.83**
Poor	60 (23.2)	131 (74.0)		
Shoe wearing to children < 3 years age				
Good	103 (39.7)	42 (23.7)	0.13 (-0.04, 0.31)	0.97
Poor	156 (60.2)	135 (76.3)		
Food hygiene at household level				
Good	130 (50.2)	25 (14.1)	0.38 (0.19, 0.56)	2.74*
Poor	129 (49.8)	152 (85.9)		
Treating household drinking water before consumption				
Good	22 (8.5)	1 (0.6)	0.08 (0.01, 0.16)	4.90**
Poor	237 (91.5)	176 (99.4)		
Regularly washing the child’s body, hands, and face				
Good	210 (81.0)	6 (3.4)	0.49 (0.38, 0.61)	15.78**
Poor	49 (19.0)	171 (96.6)		

**P < 0.001 *P < 0.05

Except for shoe wearing behavior to children under-three, a significant difference in proportions was found for almost all intervention outcomes. The proportion of respondents from the intervention woredas was about five times more likely to have appropriate practices of child feces disposal than respondents from the control woredas (t-test = 5.17; p < 0.001). Similarly, the proportion differences for the good practices of handwashing with soap or ash (t-test = 8.85; p < 0.001), maintaining a separate playground for young children (t-test = 2.83; p < 0.001), treating household drinking water (t-test = 4.90; p < 0.001), and washing a child’s body, hands, and faces (t-test = 15.78; p < 0.001) were highly significant (p < 0.001). The PSM analysis result also demonstrated a significant positive influence on good food hygiene practices (t-test = 2.74; p < 0.05). There was no significant difference between the intervention and control woredas participants in terms of shoe wearing behavior to children under-three years of age (t-test = 0.97; p > 0.05).

Findings of the qualitative assessment supported that the multimedia intervention packages and the approaches used were effective in bringing about the desired changes in the intervention implementation woredas in the Afar and Somali regions. Community members from the intervention woredas reiterated during the KIIs and FGDs with radio listener groups that improper sanitation and hygiene related practices, including poor BabyWASH practices, were detrimental before the introduction of the multimedia campaign. They stressed that open defecation was prevalent and widely practiced due to factors including low community awareness, and that communities’ awareness of the importance of BabyWASH and diarrhea prevention for child health was remarkably low.

According to the comments made by the KII and FGD participants, BabyWASH practices have improved in the intervention woredas communities in general and among women/caregivers. From their perspectives, it can be acknowledged that the multimedia educational efforts significantly contributed to changing BabyWASH and diarrhea prevention behaviors. Both personal and societal level changes in BabyWASH and diarrhea prevention practices were also indicated by participants.

*“-----Radio programs broadcast a lot of useful information in our local language [Somali]. We have come to realize the importance of maintaining a clean environment for the health of children and adults ----- I used to share the same house with our animals, but after listening to the radio program that focused on the importance of separating animals and humans shelters, I built a separate shelter for our goats-------” [KII Participant, Clan leader, Gashamo Woreda, Somali Region]*.

*“-----Young children are less likely to properly wash their hands, and they tend to get ill more easily than adults. So, it is essential to have a clean playground and equipment for babies. My child, 2.7 months old, has a separate playground with a washable mat, or “fidina” as it is known in our local community. I have also taught other mothers to do this, and most of them have already done it-----” [FGD Participant, Radio Listener Group Member, Elidar Woreda, Afar Region]*.

As well, the human interest stories gathered in the intervention woredas revealed that the intervention strategies may have effectively promoted improvements in BabyWASH practices in those woredas. A radio listener group from one of the intervention woredas in the Somali region, who is 49 and a father of eight children, was an excellent case example of how recorded radio talk show messages on memory card helped him easily disseminate messages to community members, indicating the success of the novel strategy of recording radio talk shows through memory cards to convey messages to a large segment of the target audiences.

*“------I have a habit of sharing the key messages [BabyWASH and diarrhea prevention] that are recorded and stored on the memory card in my cell phone whenever I meet people. Most of the time, those who have listened to the recorded talk show messages loved and greatly appreciated the messages, including my explanations, and they ask me to share them a copy of the recorded memory------” [Radio Listener Group Member, Gashamo Woreda, Somali Region]*.

### Potential sustainability of the intervention outcomes

Interviews with important stakeholders and community members in the intervention woredas allowed for a qualitative exploration of clues relating to the potential sustainability of changes in key BabyWASH and diarrhea prevention practices as well as the concern of whether the health system and government WASH structures will adopt the intervention efforts beyond the project term. The implementation of the intervention was participatory of existing government and community structures and this helped to gain good acceptability and sense of ownership from stakeholders according to their reflections.

*“-----The intervention [multimedia educational packages] that has been implemented in our woredas [Elidar and Afdera] for more than a year is appropriate to address the community’s WASH needs on matters relating to child health. I believe it addressed important WASH issues by implementing comprehensive educational activities in the area of BabyWASH and diarrhea prevention, paying a special focus on mothers and caregivers of babies and young children -----” [Key Informant, Afar Regional Health Bureau]*.

The multimedia educational intervention was designed and implemented with due consideration of the WASH-related policies as well as national and local WASH priorities and strategies at all levels, with the involvement of community stakeholders, such as WASH officers, HEWs, and clan leaders, which would have great significance for sustaining intervention outcomes. Also, the capacity building efforts helped to transfer knowledge and skills to key government staff and community members who play a crucial role in maintaining the intervention outcomes and initiatives. This perhaps facilitates conditions for the intervention outcomes to be sustained. The capacity building training provided to relevant stakeholders such as community members, WASH program officers, HEWs, and journalists is anticipated to strengthen efforts to enhance community awareness and practices of BabyWASH and diarrhea prevention, as highlighted by the KII participants.

*“-----I participated in the training held for HEWs in our woreda. The training was quite useful, and it encouraged each participant to actively engage in every topic raised. Using the knowledge I gained from the training, I was able to advise families on key aspects of BabyWASH during my home visits-----” [KII Participant, Health Extension Worker at Afdera Woreda, Afar Region]*.

Furthermore, the innovative and locally accepted strategy of recording radio talk shows and sharing them with community members may likely be adopted by the existing government WASH structures in order to continue conveying messages to mothers/caregivers and other members of the community. The innovative component of the intervention—using a memory card to store and record messages after the official radio broadcast—allowed radio listener groups to be refreshed with the messages and also convey them to other community members.

*“-----I have given advice to community members on issues of baby hygiene. I often play the radio in my shop and invite customers who come in for shopping, especially women, to listen to the message and engage in discussion about baby hygiene practices-----” [FGD Participant, Radio Listener Group Member, Shinile Woreda, Somali Region]*.

On the other hand, some challenges that could affect the sustainability of the intervention outcomes were identified. Essentially, poor WASH hardware infrastructures, especially the lack of access to clean water, was a rampant problem that clan leaders and community members frequently raised in the interviews. Additionally, due to COVID-19, radio listener groups were challenged to continue meeting and discussing topics related to BabyWASH and diarrhea prevention as they formerly did. It is doubtful that the radio listener groups couldn’t continue their group radio listening sessions and educating the community if there had been no follow-up mechanisms from respective local WASH structures in the target intervention woredas.

## Discussion

Population Media Center Ethiopia, in collaboration with UNICEF, has developed multimedia educational packages in project implementation hard-to-reach woredas of the Afar and Somali regions based on the UNICEF BabyWASH programming key principles [22], and this endline study was conducted to identify lessons and provide useful insights on what social and behavioral interventions can effectively promote BabyWASH and diarrhea preventive practices in these pastoralist community contexts. It is crucial to accompany BabyWASH targets with diarrhea prevention interventions to impact child health, household and community WASH outcomes, and greater environmental cleanliness [2, 20, 23]. Thus, integrating additional elements such as food hygiene and household water treatment into the BabyWASH approach helps to address the WASH needs of young children more broadly and reduce the incidence of childhood diarrheal diseases in the specific project areas.

Evidence-based behavior change interventions are important since they recognize what influences behavior change to develop interventions that can be used in specific settings [24]. Correspondingly, contextually-tailored and locally appropriate behavior change strategies are essential in BabyWASH programming [6, 17, 23]. For example, cultural concepts of play can influence the usage and appropriateness of child play spaces [17]. Key messages on BabyWASH and diarrhea prevention indicators such as safe child feces disposal, handwashing with soap or ash at critical times, protective play, shoe wearing, food hygiene, water treatment, and hygiene of children under three years old were delivered through radio talk shows and radio spots, community mobilization campaigns, and promotional print media materials in the current multimedia educational intervention. Such integration of multimedia educational interventions is suggested as an effective strategy for influencing community behavior regarding BabyWASH [25]. Moreover, a multilevel behavior change intervention that used multiple strategies such as a folk performance, transect walk, community meeting, recognition banners, and mothers’ meetings achieved marked increases in safe disposal of child feces among latrine-owning households in India [20].

The findings of the study revealed that households in the intervention woredas were more informed about key BabyWASH and diarrhea prevention practices than in the control woredas. This finding is consistent with other WASH interventions in that informational support is critical to improving household level WASH knowledge and intentions [26–28]. Qualitative data also indicated that the “radio listener group” discussion played an important role in increasing their knowledge, and such group communication approaches have been recognized as an effective medium for promoting hygiene and sanitation [25]. Similarly, the intervention had a significant impact on respondents’ beliefs and attitudes towards key BabyWASH and diarrhea prevention indicators. Findings from other studies also highlighted certain cultural norms, values and beliefs about household sanitation and hygiene practices, such as believing that fecal matter from children is not harmful and that sharing the same toilet with their children is against their beliefs [29, 30].

There was a highly significant difference in proportion between households in the intervention and control woredas on key intervention outcomes such as proper child feces disposal, handwashing with soap or ash, maintaining a separate playground for young children, treating household drinking water, and washing of a child’s body, hands, and face. The importance of educational interventions in improving household hygiene and sanitation practices has also been reported elsewhere [26, 27, 29, 31, 32]. In contrast, the evaluation highlighted that treating drinking water at the household level is practiced by a relatively small proportion of households in both the intervention and control woredas. This implies that changing behaviors through multimedia educational interventions alone may be difficult. Rather, evidence suggests that when households are provided with simple, user-friendly methods of water treatment, such as water purification chemicals/tablets, along with educational interventions, they are more likely to adopt the practice [26]. According to an endline study conducted in Northwest Ethiopia, the provision of different capacity water containers resulted in a significant change in households’ water treatment practices when compared to baseline status [31].

Our study also explored the potential long-term sustainability of changes in BabyWASH and diarrhea prevention behaviors in intervention woredas. It was indicated that the capacity building training provided to HEWs and community leaders contributed to the successful implementation of the intervention and thus the promotion of change. This is in line with other study findings, which reported that engaging community members is an important strategy for encouraging and sustaining socio-behavioral changes in sanitation and hygiene practices [33, 34]. Engaging local stakeholders, such as HEWs and community leaders, in BabyWASH through training and capacity building activities is recommended as a critical strategy for enhancing their prominent WASH-related role in the community and increasing the odds of long-term sustainability [23]. As BabyWASH behaviors are new and complex for mothers and caregivers, intensive, interpersonal visits by HEWs and the WASH structure at the grass-roots level may be necessary to promote sustainable long-term BabyWASH behavioral change [35].

Meanwhile, community members and leaders outlined low WASH infrastructure access, such as a lack of clean water, as a potential barrier to implementing BabyWASH and diarrhea prevention practices. During the formative assessment, the lack of improved sanitation facilities, and in many cases, the absence of any sanitation facilities at all, was also highlighted as a major problem in the community. This justifies the need to explore WASH facility access alongside behavior messaging in WASH interventions, particularly in rural pastoralist communities [14, 18]. Educational intervention strategies are more effective when they are combined with improved access to relevant WASH facilities (e.g., latrines, water source), and combining software and hardware WASH interventions helps to maximize intervention effectiveness [36].

This endline study used both quantitative and qualitative methods to substantiate evidence in the evaluation of the intervention’s effectiveness. We also tried to highlight potential issues and challenges regarding the long-term sustainability of the multimedia educational intervention, which is usually lacking in community WASH intervention studies. However, the study has many limitations. First, we were unable to determine the changes attributed to the intervention since the study lacked a baseline survey on pre-intervention characteristics. Second, although our formative assessment revealed no significant differences in access to water and sanitation facilities between the intervention and control woredas, specific comparative data on levels of water and sanitation access was not gathered from survey respondents to provide more context to the study findings. Third, the duration of the intervention was short (1.8 months) to evaluate long-term effects in the retention of intervention outcomes. Fourth, while control woredas with comparable sociodemographic and living condition were selected, there may be a risk of selection bias due to the non-random selection of the intervention woredas for the implementation of the multimedia educational intervention. Fifth, given that the data was collected during the COVID-19 pandemic, some of the survey responses may have been influenced by WASH-related Covid-19 messages, such as messages to regularly wash hands. The COVID-19 pandemic had also affected how FGDs were conducted, as the rule of eight to ten participants per FGD wasn’t followed. Due to social gathering restrictions, we limited the number of FGD participants to a maximum of four and kept social distance during the discussions. Lastly, we acknowledge that the primary outcome is subject to response bias as it is measured using caregiver self-report, especially when the “usual” practice is asked rather than the “last time” practice.

## Conclusion

This endline evaluation demonstrated that the multimedia educational intervention improved awareness, perception, and practice of BabyWASH and diarrhea prevention behaviors in intervention woredas compared to control woredas. The achievements of the project can help to inform the design and implementation of future social and behavior change interventions to improve hygiene and sanitation practices for children under three years old, as well as household-level behavioral changes for prevention of child diarrheal diseases. This endline evaluation especially gives insight that locally and contextually appropriate intervention strategies, such as the use of “radio listener groups” and the involvement of community stakeholders, can be effective strategies in sanitation and hygiene promotion interventions in rural pastoralist settings. However, issues about the long-term sustainability of the BabyWASH and diarrhea prevention outcomes have been recognized as an allusive concern, and revisiting project areas may provide substantive evidence to assess the long-term sustainability of intervention outcomes. In future WASH interventions, considerations for integrating both behavioral and structural components are also essential for increased gains from social and behavior change interventions targeting WASH behaviors. Additionally, as BabyWASH is a new concept, more research evidence based on context- and culture-dependent social and behavior change approaches is needed to improve BabyWASH and household diarrhea prevention practices, particularly in rural pastoralist communities.

### Electronic supplementary material

Below is the link to the electronic supplementary material.

Additional file 1: Examples of BabyWASH radio talk show and radio spot messages

Additional file 2: Measurement cut-off points to operationally categorize BabyWASH and diarrhea prevention practices as “good” or “poor”

## Data Availability

The datasets generated and/or analysed during the current study are available from the corresponding author upon reasonable request.

## Abbreviations

FGD: Focus group discussion
HEW: Health Extension Worker
KII: Key-informant interview
LMICs: Low- and middle-income countries
PSM: Propensity score matching
UNICEF: United Nations Children’s Fund
WASH: Water, sanitation, and hygiene

## References

[1] Ngure FM, Reid BM, Humphrey JH, Mbuya MN, Pelto G, Stoltzfus RJ (2014). Water, sanitation, and hygiene (WASH), environmental enteropathy, nutrition, and early child development: making the links. Ann N Y Acad Sci.

[2] Macintyre A, Strachan C, Sanitation, Hygiene and Environmental Cleanliness for Child Development. Brighton IDS: Frontiers of Sanitation: Innovations and Insights 19; 2021. https://opendocs.ids.ac.uk/opendocs/handle/20.500.12413/16954. Accessed 07 Nov 2022.

[3] Waller A, Lakhanpaul M, Godfrey S, Parikh P (2020). Multiple and complex links between babyWASH and stunting: an evidence synthesis. J Water Sanitation Hygiene Dev.

[4] Ngure F (2012). Environmental, Hygiene, Food Safety and Growth in less than five Year Old Children in Zimbabwe and Ethiopia.

[5] Troeger C, Blacker BF, Khalil IA, Rao PC, Cao S, Zimsen SR (2018). Estimates of the global, regional, and national morbidity, mortality, and aetiologies of diarrhoea in 195 countries: a systematic analysis for the global burden of disease study 2016. Lancet Infect Disease.

[6] Federal Ministry of Health of Ethiopia. Baby and, Mother WASH, Implementation. Guideline; 2017. https://www.unicef.org/ethiopia/media/1236/file/Baby%20and%20Mother%20WASH%20Implementation%20Guideline.pdf. Accessed 07 Nov 2022.

[7] Alebel A, Tesema C, Temesgen B, Gebrie A, Petrucka P, Kibret GD (2018). Prevalence and determinants of diarrhea among under-five children in Ethiopia: a systematic review and meta-analysis. PLoS ONE.

[8] United Nations Children’s Fund (UNICEF) and World Health Organization (WHO) (2019). Progress on household drinking water, sanitation and hygiene 2000–2017: special focus on inequalities.

[9] Bauza V, Guest JS (2017). The effect of young children’s faeces disposal practices on child growth: evidence from 34 countries. Trop Med Int Health.

[10] Biniyam S. Prevalence and associated factors of safe and improved infant and young children stool disposal in Ethiopia: Evidence from demographic and health survey. *BMC Public Health* 2019; 19(970). 10.1186/s12889-019-7325-9 PMC6647302 31331313

[11] Mugel SG, Clasen TF, Bauza V. Global practices, geographic variation, and determinants of child feces disposal in 42 low- and middle-income countries: An analysis of standardized cross-sectional national surveys from 2016–2020. *Int J Hyg Environ Health* 2022; 245(114024). 10.1016/j.ijheh.2022.114024 PMC9489922 36029740

[12] Fischer Walker CL, Perin J, Aryee MJ, Boschi-Pinto C, Black RE. Diarrhea incidence in low- and middle-income countries in 1990 and 2010: a systematic review. BMC Public Health 2012; 12(220). 10.1186/1471-2458-12-220 PMC3323412 22436130

[13] Majorin F, Torondel B, Ka Seen Chan G, Clasen T (2019). Interventions to improve disposal of child faeces for preventing diarrhoea and soil-transmitted helminth infection. Cochrane Database Syst Rev.

[14] Whitley L, Hutchings P, Cooper S, Parker A, Kebede A (2019). A framework for targeting water, sanitation and hygiene interventions in pastoralist populations in the Afar region of Ethiopia. Int J Hyg Environ Health.

[15] Bekele T, Rawstorne P, Rahman B (2020). Effect of water, sanitation and hygiene interventions alone and combined with nutrition on child growth in low and middle income countries: a systematic review and meta-analysis. BMJ Open.

[16] Pickering AJ, Null C, Winch PJ, Mangwadu G, Arnold BF, Prendergast AJ (2019). The WASH benefits and SHINE trials: interpretation of WASH intervention effects on linear growth and diarrhoea. Lancet Glob Health.

[17] Budge S, Parker A, Hutchings P, Garbutt C, Rosenbaum J, Tulu T (2021). Multi-sectoral Participatory Design of a BabyWASH Playspace for Rural Ethiopian Households. Am J Trop Med Hyg.

[18] Bitew BD, Woldu W, Gizaw Z. Childhood diarrheal morbidity and sanitation predictors in a nomadic community. Ital J Pediatr 2017; 43(91). 10.1186/s13052-017-0412-6 PMC5639577 28985750

[19] Gizaw Z, Woldu W, Bitew BD. Child feeding practices and diarrheal disease among children less than two years of age of the nomadic people in Hadaleala District, Afar Region, Northeast Ethiopia. *Int Breastfeed J* 2017; 12(24). 10.1186/s13006-017-0115-z PMC5460459 28592985

[20] Caruso BA, Sclar GD, Routray P, Nagel CL, Majorin F, Sola S (2022). Effect of a low-cost, behaviour-change intervention on latrine use and safe disposal of child faeces in rural Odisha, India: a cluster-randomised controlled trial. Lancet Planet Health.

[21] Tolley EE, Ulin PR, Mack N, Robinson ET, Succop SM. Qualitative methods in Public Health: a Field Guide for Applied Research. 2nd ed. Jossey-Bass; 2016.

[22] UNICEF, Baby WASH, Programming. Integrating water, sanitation and hygiene interventions across sectors to impact child health outcomes; 2020. https://www.unicef.org/esa/media/7076/file/UNICEF-ESA-Baby-WASH-Programming-2020.pdf. Accessed 07 Nov 2022.

[23] Action Against Hunger. Implementing a BabyWASH approach: lessons learned from conflict-affected pregnant and lactating women in Yobe, Nigeria. Water, Sanitation & Hygiene Nutrition & Health Endline BabyWASH Report;. 2019. https://www.fsnnetwork.org/sites/default/files/BabyWASH_Report_Nigeria_2019_final.pdf. Accessed 07 Nov 2022.

[24] Dreibelbis R, Winch PJ, Leontsini E, Hulland KR, Ram PK et al. The Integrated Behavioural Model for Water, Sanitation, and Hygiene: a systematic review of behavioural models and a framework for designing and evaluating behaviour change interventions in infrastructure-restricted settings. *BMC Public Health* 2013; 13(1015). 10.1186/1471-2458-13-1015 PMC4231350 24160869

[25] Sriram A, Maheswari U. Integrated communication strategy for creating awareness on sanitation and hygiene behavior change. Int J Commun Health 2013; 14(1).

[26] Phuanukoonnon S, Namosha E, Kua L, Siba PM, Greenhill AR (2013). Evaluation of a WASH intervention demonstrates the potential for improved hygiene practices in Hiri District, Central Province. P N G Med J.

[27] Fisher S, Kabir B, Lahiff E, Maclachlan M (2011). Knowledge, attitudes, practices and implications of safe water management and good hygiene in rural Bangladesh: assessing the impact and scope of the BRAC WASH programme. J Water Health.

[28] Sclar GD, Bauza V, Bisoyi A, Clasen TF (2022). Contextual and psychosocial factors influencing caregiver safe disposal of child feces and child latrine training in rural Odisha, India. PLoS ONE.

[29] Hetherington E, Eggers M, Wamoyi J, Hatfield J, Manyama M et al. Participatory science and innovation for improved sanitation and hygiene: process and outcome evaluation of project SHINE, a school-based intervention in Rural Tanzania. BMC Public Health 2017; 17(172). 10.1186/s12889-017-4100-7 PMC5297194 28173789

[30] Shire BA, Nzioki JM, Mambo S, Muhamud C, Korir A (2020). Socio-Cultural factors influencing child faecal matter disposal among caregivers in Wadajir District in Mogadishu-Somalia. Afr J Health Sci.

[31] Gizaw Z, Addisu A. Evidence of Households’ Water, Sanitation, and Hygiene (WASH) Performance Improvement Following a WASH Education Program in Rural Dembiya, Northwest Ethiopia. Environ Health Insights 2020; 14(1178630220903100). 10.1177/1178630220903100 PMC7003158 32076370

[32] Schmidt WP, Cairncross S (2009). Household water treatment in poor populations: is there enough evidence for scaling up now?. Environ Sci Technol.

[33] Crocker J, Saywell D, Bartram J (2017). Sustainability of community-led total sanitation outcomes: evidence from Ethiopia and Ghana. Int J Hyg Environ Health.

[34] Ashwell HE, Barclay L (2009). Outcome evaluation of community health promotion intervention within a donor funded project climate in Papua New Guinea. Rural Remote Health.

[35] USAID and Centre for Diarrhoeal Disease Research, Bangladesh. Using Mobile Health Messaging to Nudge Babywash Behavior: Formative Research Final Report. Washington, DC: USAID. Water, Sanitation, and Hygiene Partnerships and Learning for Sustainability (WASHPaLS) Project. ; 2020. https://www.globalwaters.org/sites/default/files/icddrb_mhealth_final_grant_report.pdf. Accessed 07 Nov 2022.

[36] Research LS. Formative Research on Wash, Child Health and Nutrition. The World Bank. https://thedocs.worldbank.org/en/doc/511861594659959192-0090022020/render/LaoPDRTF0A9471BabyWaSHFinalReport8decclean.pdf. Accessed 07 Nov 2022.

