# Supplementary material for: BabyWASH and diarrhea prevention practices following multimedia educational intervention in hard-to-reach areas of the Afar and Somali regions of Ethiopia: a mixed-method endline evaluation
Source: BMC Public Health. 2023 Oct 13;23:1998. doi: 10.1186/s12889-023-16887-y (PMC10576324; doi:10.1186/s12889-023-16887-y)
Supplement: Supplementary file 1 — Additional file 1: Examples of BabyWASH radio talk show and radio spot messages [file 12889_2023_16887_MOESM1_ESM.docx]

**Additional file 1:** Examples of BabyWASH radio talk show and radio spot messages

Proper disposal of child feces

- All feces, including those of babies and young children, should be disposed of in a latrine.
- Proper disposal of child feces in a latrine prevent the spread of diarrheal diseases.
- Throw all feces including baby’s feces into a latrine. Where there is no latrine feces should be buried.

Handwashing with soap or ash

- You and your family should wash your hands frequently with soap or ash to prevent diseases such as diarrhea.
- Always wash your hand with soap or ash before preparing foods.
- Wash your hand with soap or ash before feeding the baby.
- Wash your hands with soap or ash after using the toilet or before cleaning a baby’s bottom.

Playground for young children

- Preparing a separate playground for children is important to prevent contamination with animal waste and other environmental risks, which can cause diarrheal diseases.
- Playground should be safe and clean to reduce the risk of injury and accident.

Child hygiene

- Washing children’s hands, face and body regularly is important for their healthy development.
- Washing the face and hands with soap every day helps to prevent eye infections which can lead to trachoma, which can cause blindness.

Wearing shoes for children

- Wearing shoes for babies helps to protect their feet from injury and the heat.

Water treatment at home

- Drinking water sources may contain a variety of contaminants that are particularly hazardous for young children.
- Boil or treat all drinking water at home before consumption to ensure it is safe.

Food hygiene

- Wash your hand before handling food.
- Wash all the equipment and utensils used for food preparation.
- Avoid contact between raw and prepared foods.
- Cook food thoroughly, especially meat and egg.
