# Supplementary material for: BabyWASH and diarrhea prevention practices following multimedia educational intervention in hard-to-reach areas of the Afar and Somali regions of Ethiopia: a mixed-method endline evaluation
Source: BMC Public Health. 2023 Oct 13;23:1998. doi: 10.1186/s12889-023-16887-y (PMC10576324; doi:10.1186/s12889-023-16887-y)
Supplement: Supplementary file 2 — Additional file 2: Measurement cut-off points to operationally categorize BabyWASH and diarrhea prevention practices as “good” or “poor” [file 12889_2023_16887_MOESM2_ESM.docx]

**Additional file 2:** Measurement cut-off points to operationally categorize BabyWASH and diarrhea prevention practices as “good” or “poor”

- ***Child feces disposal:*** Participants were asked, “Where did you dispose of the child feces the last time the child passed stool?” Participants who reported disposing of child feces in a latrine or buried were classified as having “good” child feces disposal practices, whereas those who reported disposing of child feces into a garbage pit or throwing it away in the backyard were classified as having “poor” child feces disposal practices.
- ***Handwashing practices at critical times with soap/ash:*** A multiple answer question “When do you usually wash your hands?” was asked to survey participants with observation of a handwashing device with soap/ash designated in the nearby toilets as supporting evidence. The practice was considered “good” if respondents specified at least two of the five critical times of handwashing with soap/ash (i.e., after using latrine, after cleaning baby bottom, before eating, preparing food and breastfeeding). However, specifying less than two of the five critical times of handwashing with soap/ash was considered as having “poor” handwashing practices at critical times. In addition, if a handwashing facility was not available in the household at the time of data collection, the practice was classified as “poor” regardless of the response to the interview question.
- ***Protective play:*** Households that reported maintaining a separate playground for their children under three years of age and provided supportive evidence during observation of the type of playground materials (such as cleanable mats) were categorized as having “good” practice, while households that reported not maintaining a separate playground for their children under three years of age were categorized as having “poor” practice.
- ***Shoe wearing behavior:*** Participants were asked “Do you frequently wear shoes for your child under three years?” and observations were made to determine whether young children were wearing shoes. Mothers/caregivers who reported that their child wear shoes and the child was actually seen wearing shoe at the time of data collection were considered as engaging in “good” practice.
- ***Food hygiene:*** To assess food hygiene practices, participants were asked “How do you prepare food for you and your children in the household?” without offering any optional choices of good hygienic food preparation steps. Those respondents who mentioned all the good ways of food preparation in the households—including hand and utensil washing, safe storage, and reheating of cooked food—were considered to have “good” food hygiene practices.
- ***Water treatment:*** Households that reported treating water before drinking—either through filtration, boiling or chlorination—were classified as having “good” practices, while those that reported not doing so were classified as having “poor” practices.
- ***Child hygiene:*** Participants were asked “How frequently do you wash your child's body, hands, and face each day?” with response options of “once every day”, “twice per day”, “three times per day”, “whenever it is necessary,” and “not every day”. Mothers and caregivers who reported washing their child’s body, hands, and face at least once a day were considered to have “good” practices. In contrast, those who reported not washing their child’s body, hands and face every day were considered to have “poor” practices.
